# Systems analysis and improvement approach to optimize the hypertension diagnosis and case cascade for PLHIV individuals (SAIA-HTN): a hybrid type III cluster randomized trial

**DOI:** 10.1186/s13012-020-0973-4

**Published:** 2020-03-06

**Authors:** Sarah Gimbel, Ana Olga Mocumbi, Kristjana Ásbjörnsdóttir, Joana Coutinho, Leonel Andela, Bonifacio Cebola, Heidi Craine, Jonny Crocker, Leecreesha Hicks, Sarah Holte, Rodrigues Hossieke, Edgar Itai, Carol Levin, Nelia Manaca, Filipe Murgorgo, Miguel Nhumba, James Pfeiffer, Isaias Ramiro, Keshet Ronen, Nona Sotoodehnia, Onei Uetela, Anjuli Wagner, Bryan J. Weiner, Kenneth Sherr

**Affiliations:** 1 Department of Child, Family and Population Health Nursing, University of Washington School of Nursing, 1959 NE Pacific St, Seattle, WA 98195 USA; 2 Department of Global Health, University of Washington Schools of Medicine and Public Health, 1705 NE Pacific St, Seattle, WA 98195 USA; 3 Health Alliance International (HAI), 1107 NE 45th St, Suite 350, Seattle, WA 98105 USA; 4 Faculty of Medicine, Eduardo Mondlane University, Avenida Salvador Allende, 702 Maputo, Mozambique; 5 Health Alliance International, Caixa Postal, #23 Maputo, Mozambique; 6 Department of Epidemiology, University of Washington School of Public Health, 1705 NE Pacific St, Seattle, WA 98195 USA; 7 Beira Central Hospital, Beira, Mozambique; 8 Manica Provincial Health Department, Chimoio, Mozambique; 9 Sofala Provincial Health Department, Beira, Mozambique; 10 Department of Cardiology, University of Washington School of Medicine, 325 9th Avenue, Seattle, WA 98104 USA

**Keywords:** Systems analysis and improvement approach (SAIA), Hypertension, CFIR, ORIC, Process mapping, Cascade analysis, Continuous quality improvement, Implementation science, Systems engineering, HIV

## Abstract

**Background:**

Across sub-Saharan Africa, evidence-based clinical guidelines to screen and manage hypertension exist; however, country level application is low due to lack of service readiness, uneven health worker motivation, weak accountability of health worker performance, and poor integration of hypertension screening and management with chronic care services. The systems analysis and improvement approach (SAIA) is an evidence-based implementation strategy that combines systems engineering tools into a five-step, facility-level package to improve understanding of gaps (cascade analysis), guide identification and prioritization of low-cost workflow modifications (process mapping), and iteratively test and redesign these modifications (continuous quality improvement). As hypertension screening and management are integrated into chronic care services in sub-Saharan Africa, an opportunity exists to test whether SAIA interventions shown to be effective in improving efficiency and coverage of HIV services can be effective when applied to the non-communicable disease services that leverage the same platform. We hypothesize that SAIA-hypertension (SAIA-HTN) will be effective as an adaptable, scalable model for broad implementation.

**Methods:**

We will deploy a hybrid type III cluster randomized trial to evaluate the impact of SAIA-HTN on hypertension management in eight intervention and eight control facilities in central Mozambique. Effectiveness outcomes include hypertension cascade flow measures (screening, diagnosis, management, control), as well as hypertension and HIV clinical outcomes among people living with HIV. Cost-effectiveness will be estimated as the incremental costs per additional patient passing through the hypertension cascade steps and the cost per additional disability-adjusted life year averted, from the payer perspective (Ministry of Health). SAIA-HTN implementation fidelity will be measured, and the Consolidated Framework for Implementation Research will guide qualitative evaluation of the implementation process in high- and low-performing facilities to identify determinants of intervention success and failure, and define core and adaptable components of the SAIA-HTN intervention. The Organizational Readiness for Implementing Change scale will measure facility-level readiness for adopting SAIA-HTN.

**Discussion:**

SAIA packages user-friendly systems engineering tools to guide decision-making by front-line health workers to identify low-cost, contextually appropriate chronic care improvement strategies. By integrating SAIA into routine hypertension screening and management structures, this pragmatic trial is designed to test a model for national scale-up.

**Trial registration:**

ClinicalTrials.gov NCT04088656 (registered 09/13/2019; https://clinicaltrials.gov/ct2/show/NCT04088656).

Contributions to the literature

- Our study examines whether introduction of a user-friendly, low-cost package of systems engineering tools delivered iteratively by trained mentors through health facility teams in a low-income, public sector health system improves health system delivery of hypertension care and related patient outcomes.
- Our study is designed to provide decision-makers with the evidence they need to decide whether and how to further scale-up the approach, specifically through targeted guidance on implementation across heterogeneous settings as well as costs of this approach.
- Research on systems engineering tools has largely come from high-resourced health systems, with little data from low-income countries.

Contributions to the literature

- Our study examines whether introduction of a user-friendly, low-cost package of systems engineering tools delivered iteratively by trained mentors through health facility teams in a low-income, public sector health system improves health system delivery of hypertension care and related patient outcomes.
- Our study is designed to provide decision-makers with the evidence they need to decide whether and how to further scale-up the approach, specifically through targeted guidance on implementation across heterogeneous settings as well as costs of this approach.
- Research on systems engineering tools has largely come from high-resourced health systems, with little data from low-income countries.

## Background

Hypertension is the leading risk factor for death globally and is more prevalent in sub-Saharan Africa (sSA) [1]. A systematic review across the region reported hypertension prevalence between 15 and 70% (median prevalence 29%) [2]. Yet most hypertension in sSA is undiagnosed and untreated, further worsening the region’s rising burden of cardiovascular disease [3–5]. In Mozambique (a low-income country with > 13% adult HIV prevalence) [6], hypertension prevalence among adults 25–64 years increased significantly from 2005 to 2015, now affecting nearly 40% of adults [7].

Human immunodeficiency virus (HIV) and hypertension comorbidity is common and increasing, in large part due to an aging people living with HIV (PLHIV) population and side effects of antiretroviral therapy (ART). Survivorship among PLHIV has increased due to improvements in ART access and effectiveness, thus expanding the number of older PLHIV, who are more likely to experience comorbid hypertension [8–12]. The prevalence of hypertension in PLHIV is estimated to range from 9 to 46% in low- and middle-income countries (LMICs) [13, 14], similar to high-income countries [15–17]. Patients on ART are more likely to have dysglycemia, hypertriglyceridemia, and lower levels of high-density lipoprotein cholesterol [18–20], which may increase hypertension risk. Also relevant, studies indicate that HIV may increase the risk of non-communicable disease (NCD), including cardiovascular disease CVD, due to stimulation of inflammatory markers and adverse events of ART [21–24].

Across sSA (including Mozambique), efficacious, low-cost, and safe hypertension (HTN) screening and treatment options exist, and evidence-based clinical guidelines for hypertension management have been developed and disseminated by the Pan-African Society of Cardiology (PASCAR) that recommend routine screening and management of adult PLHIV for CVD risk factors [25]. Yet guideline application is low due to a lack of service readiness (e.g., availability of essential equipment, health worker training), uneven health worker motivation, weak accountability of health worker performance, and poor integration of hypertension screening and management within chronic care services [26]. As a result, self-reported knowledge of blood pressure status and treatment coverage is low. Less than half of individuals with hypertension in sSA are aware of their condition [27]. In Mozambique, less than 15% of hypertensive adults are aware of their hypertension status (the lowest in sSA); among these, only 50% are in treatment, and less than half of whom have controlled hypertension [7]. As a result of this leaky hypertension cascade, only 3% of the total adult population with hypertension in Mozambique have their condition controlled.

Approaches to optimize care cascades are needed to maximize the benefits of hypertension screening and management for PLHIV [28]. Barriers to hypertension diagnosis and management for PLHIV exist on the individual (patient and/or caregiver) [26, 29], interpersonal (provider) [30, 31], health systems [32], and policy levels [33]. Existing research emphasizes individual and interpersonal-level barriers; however, *health systems* barriers receive less attention [34], presenting an opportunity to improve patient health by optimizing the complex hypertension cascade. The hypertension cascade presents a series of linked steps (screening, diagnosis, management, and control), whereby achieving improved patient outcomes is conditional on successful progression through all service steps. Robust systems engineering methods (e.g., cascade analysis, process mapping, and continuous quality improvement) can optimize poorly functioning cascades by (1) identifying main drivers for system inefficiency, (2) supporting locally informed provider decision-making to prioritize intervention, and (3) improving integration of services to meet patient needs across diverse chronic care contexts [28, 35]. Low-cost, systems-level interventions are effective and efficient approaches to improve linked cascade services, and may be effective for routinizing hypertension diagnosis and management within existing ambulatory services; addressing both individual and system-level barriers; improving flow through the hypertension care cascade; and ultimately improving patient-level outcomes.

The systems analysis and improvement approach (SAIA) is designed to optimize cascade performance, is feasible for frontline healthcare workers and managers, and may be applicable to optimize the hypertension testing and treatment cascade for PLHIV across multiple contexts. SAIA, an evidence-based strategy, which is flexible to local context, supports frontline facility staff to gain a comprehensive view of their complex delivery system, identify and prioritize areas to improve, and iteratively test modifications to increase system outputs and patient outcomes [36, 37]. Health workers in the original cluster randomized trial that tested the intervention to improve prevention of mother-to-child HIV transmission (PMTCT) services noted that SAIA stimulated communication, consensus decision-making, and accountability across multiple service points in their facility (e.g., patient care, pharmacy, laboratory services); was accessible by relying on routinely collected data to guide decision-making in a real-world service delivery environment; and resulted in significant improvements in service delivery outcomes [37]. SAIA for the hypertension cascade for PLHIV (SAIA-HTN) was successfully piloted in outpatient services in the study area over a 3-month period, and demonstrated a 31% increase in hypertension screening [38]. Given the current health service delivery challenges and the promising pliot results, ascertaining whether SAIA-HTN is an effective, adaptable, and scalable model for broad implementation is scientifically important.

### Goals and objectives

The overall goal of this study is to evaluate a model for systematic assessment and improvement of hypertension diagnosis and management services for PLHIV individuals in Mozambique (SAIA-HTN). Over the 5-year project, the investigators will conduct a facility-level parallel hybrid type III cluster randomized trial [39] to evaluate SAIA-HTN effectiveness on hypertension cascade optimization (aim 1); identify drivers of implementation success (aim 2); and determine costs and cost effectiveness on hypertension and HIV clinical measures (aim 3). Our design has a number of advantages for this study including (1) it will assess SAIA-HTN’s performance under pragmatic conditions, (2) a randomized design reduces potential confounding and will be able to provide robust evidence of SAIA-HTN’s impact, and (3) it facilitates assessment of maturation patterns in SAIA-HTN’s effect over time, during both the intensive and sustainment phases.

## Methods

### Description of the SAIA-HTN implementation strategy

**Fig. 1:**
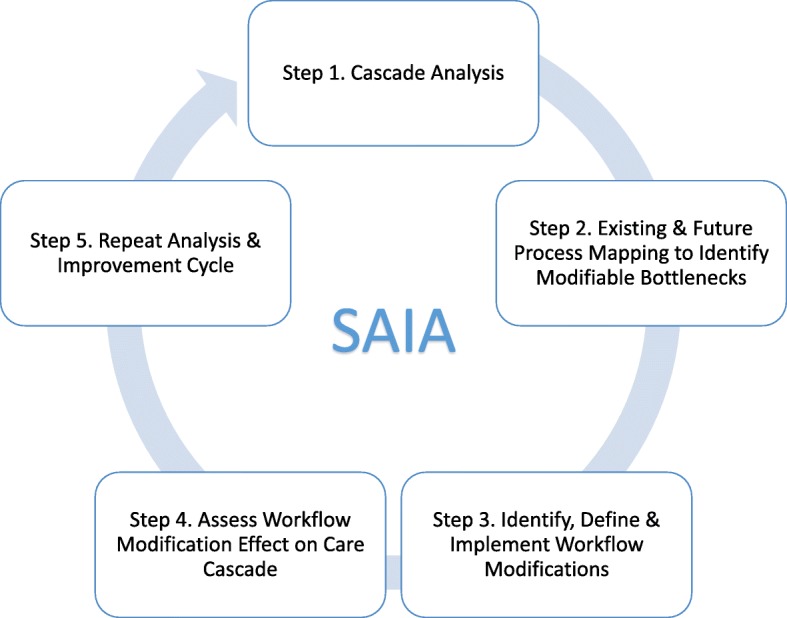
The systems analysis and improvement approach (SAIA)

**Fig. 2:**
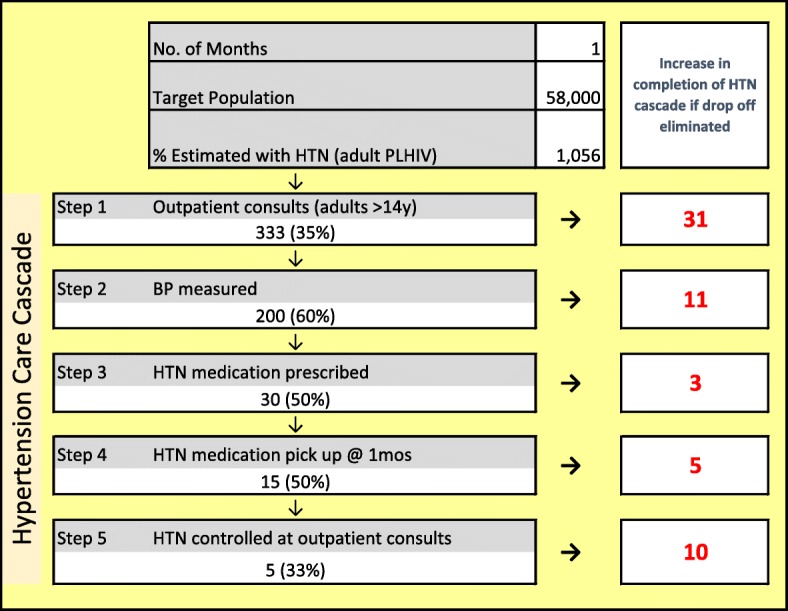
Hypertension cascade analysis tool (HCAT) for people living with HIV

SAIA-HTN packages systems engineering tools into an iterative, five-step process applied at the facility level to give clinic staff and managers a system-wide view of their cascade performance, identify priority areas for improvement, discern modifiable opportunities for improvement, and test these workflow modifications (Fig. 1). SAIA has been previously described in the literature [40, 41]. In brief, in its adapted form, the SAIA-HTN cascade includes the following steps:

- Step 1: Understand targeted cascade performance, and identify priority areas for improvement. The HTN cascade analysis tool (HCAT) (Fig. 2) uses routine data to provide a rapid, systems-level view of drop-offs along the hypertension cascade for PLHIV, with an optimization function that allows the user to rapidly assess how many additional people will be served if only one step is fully optimized while other stay the same [28]. As an analytic tool, HCAT helps frontline staff and facility managers to prioritize where to intervene by providing a view of the greatest potential for flow improvements across the entire cascade.
- Step 2: Process mapping to identify facility-level modifiable bottlenecks. Enabling facility-level staff to identify and gain consensus on bottlenecks to address in their hypertension system is essential to defining innovations to implement. SAIA-HTN applies sequential process flow mapping procedures [35, 42], coupled with workflow observation, to identify bottlenecks and guide discussion on opportunities for workflow modifications.
- Step 3: Define and implement facility-specific workflow adaptations to address modifiable bottlenecks. After identifying modifiable barriers within cascade steps, facility staff identify a simple, specific change to improve performance within the targeted step. Selected workflow adaptations should be feasible to implement, be within the scope of influence of facility management and frontline staff, and be expected to lead to rapid, substantial improvements in the targeted cascade step. Ideas for adaptations come from brainstorming solutions with facility staff, complemented by best practices from the literature and high performing services in Mozambique. An implementation plan for the innovation is described in writing by facility and study personnel to ensure consensus among facility staff, and clarify operational design and roles. Steps 3 and 4 are analogous to continuous quality improvement.
- Step 4: Monitor changes in routine performance. Facility staff monitor change in routinely reported data from the cascade step selected for improvement. Measuring the absolute change in the proportion of patients progressing through targeted steps captures large, rapid improvements accompanying modifications.
- Step 5: Repeat cycle. Systems engineering improvement processes are by definition iterative, with ongoing testing of innovations responsive to evolving, contextually specific barriers. Facility staff repeat steps 1–5 at the end of each cycle to identify new approaches to modify previously identified barriers, or if the first cycle was successful, focusing on improving priority bottlenecks identified in a repeated systems analysis.

### SAIA-HTN trial design

Using a 3-year parallel hybrid type III cluster randomized controlled design, we will prospectively implement SAIA-HTN and evaluate its impact for PLHIV individuals in eight intervention and eight control facilities in central Mozambique (CONSORT Checklist, Additional file 1). Public sector district HIV and non-communicable disease (NCD) supervisors, supported by study nurses, will deliver the intervention over a 2-year intensive phase, followed by a 1-year sustainment phase led by the district HIV and NCD supervisors without additional study personnel support, which will provide evidence on intervention impact under ideal circumstances, as well as the longevity of the effect over time (Table 1). The mixed-methods evaluation will evaluate the impact of SAIA-HTN on clinical as well as process outcomes (Table 2). The organizational readiness for implementing change (ORIC) will be applied to facilities earlier in SAIA-HTN implementation (within 3 months of starting) to identify organizational-level attributes that affect intervention adoption. The Consolidated Framework for Implementation Research (CFIR) will be used to guide data collection and interpretation related to implementation, to assess fidelity to intervention protocol, describe intervention adaptations when integrated into routine management systems [43], and identify organizational-level determinants of successful SAIA-HTN implementation. In addition, recurrent measurement of structural readiness and implementation dose (as a function of quality and quantity) at the facility level will inform guidance on essential structural needs to implement SAIA-HTN. Based on trial results, we will model the costs and benefits on hypertension control and HIV viral suppression given different scale-up scenarios within Mozambique. The trial will culminate in a dissemination package, summarizing trial results and providing implementation and cost guidance to support national SAIA-HTN scale-up.

**Table 1:**
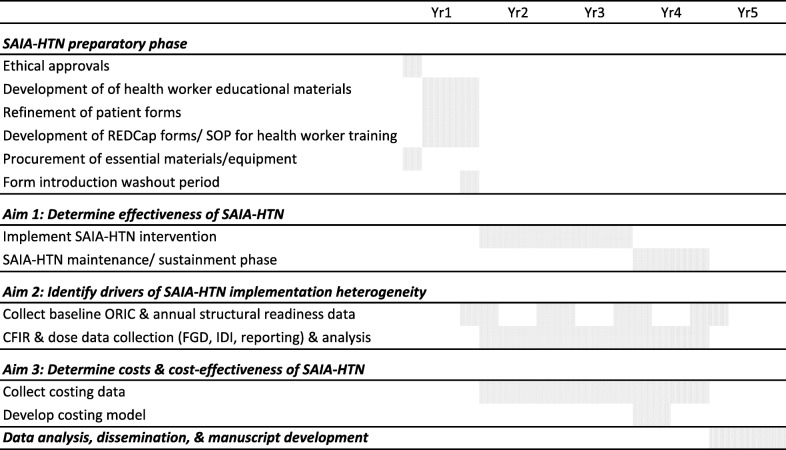
Study timeline

Key. *SAIA-HTN*: Systems Analysis and Improvement Approach for the Hypertension Care, Cascade; *REDcap*: a secure web application for building and managing online surveys and databases; *SOP*: Standard Operating Procedures; *ORIC*: Organizational Readiness for Implementing Change; *CFIR*: Consolidated Framework for Implementing Change; *FGDs*: Focus Group Discussions; *IDI*: In-depth Interviews

**Table 2:** SAIA-HTN study primary and secondary outcomes

Type	Indicator	Numerator	Denominator
Process	BP screening*	**# PLHIV in ambulatory care consults screened for BP**	**# Ambulatory care consults for PLHIV**
	HTN Diagnosis	# PLHIV with new or existing HTN diagnosis in ambulatory care consults	# PLHIV in ambulatory care consults screened for BP
	HTN Medication prescribed	# PLHIV with new or existing HTN diagnosis prescribed HTN medication	# PLHIV with new or existing HTN diagnosis in ambulatory care consults
	HTN Medication pick up	# PLHIV prescribed HTN medications who picked up their medications the previous month	# PLHIV with new or existing HTN diagnosis prescribed HTN medication
Clinical	Controlled HTN amongst those on treatment*	**# PLHIV with new or existing HTN diagnosis and taking HTN medications who have controlled HTN**	**# PLHIV prescribed HTN medications who picked up their medications the previous month**
	Undetectable HIV viral load	# PLHIV with HTN and a non-detectable HIV viral load	# PLHIV with HTN

Bolded and *= primary outcome; *BP* blood pressure, *PLHIV* people living with HIV, *HIV* human immunodeficiency virus, *HTN* hypertension

### Process for introducing SAIA

SAIA-HTN’s standard operating procedures (SOPs), including the delivery and training schedules as well as the intervention guides and tools (HCAT, process mapping, and CQI guides), were developed during the initial SAIA-HTN pilot and will be refined in preparation for the SAIA-HTN trial. In the 3 months prior to initiation of the intensive phase, facility teams (including managers and staff from ambulatory and pharmacy services) receive a 5-day orientation from study teams and district health authorities on using the SAIA-HTN SOPs, including an introduction to the SAIA tools, the implementation schedule, and the data collection procedures. During this first week of SAIA-HTN implementation, facility teams also populate and interpret the HCAT, develop process maps of current PLHIV patient care pathways to hypertension diagnosis and management, define micro-interventions, and indicators to monitor these modifications.

Subsequently, district non-communicable disease (NCD) and HIV supervisors, together with study nurses, introduce SAIA-HTN to intervention health facilities over a 2-day period. Within each district, SAIA-HTN is introduced one facility at a time, until all intervention facilities are covered in each district. Facility teams receive bi-weekly supervision visits by study personnel and district supervisors for the first month, followed by monthly visits throughout the remainder of the 24-month intensive implementation period. During the third year after SAIA-HTN introduction (sustainment), monthly mentorship visits will be conducted by district authorities without support from study personnel to evaluate SAIA-HTN sustainability with moderate resource investment (including transport to facilities and per diem for staff).

Based on the SAIA trial and SAIA-HTN pilot, it is expected that analysis and improvement cycles will occur monthly, with an average of 12 cycles per year per facility. A SAIA core component that will be maintained in SAIA-HTN is provision to intervention facilities of a flexible facility support fund to address basic equipment needs for hypertension management (sphygmomanometer, scales, stethoscope, etc.), as well as to support workflow modifications, which will continue throughout both the intervention and sustainment periods.

### Study setting and eligibility criteria

#### Study setting

**Fig. 3:**
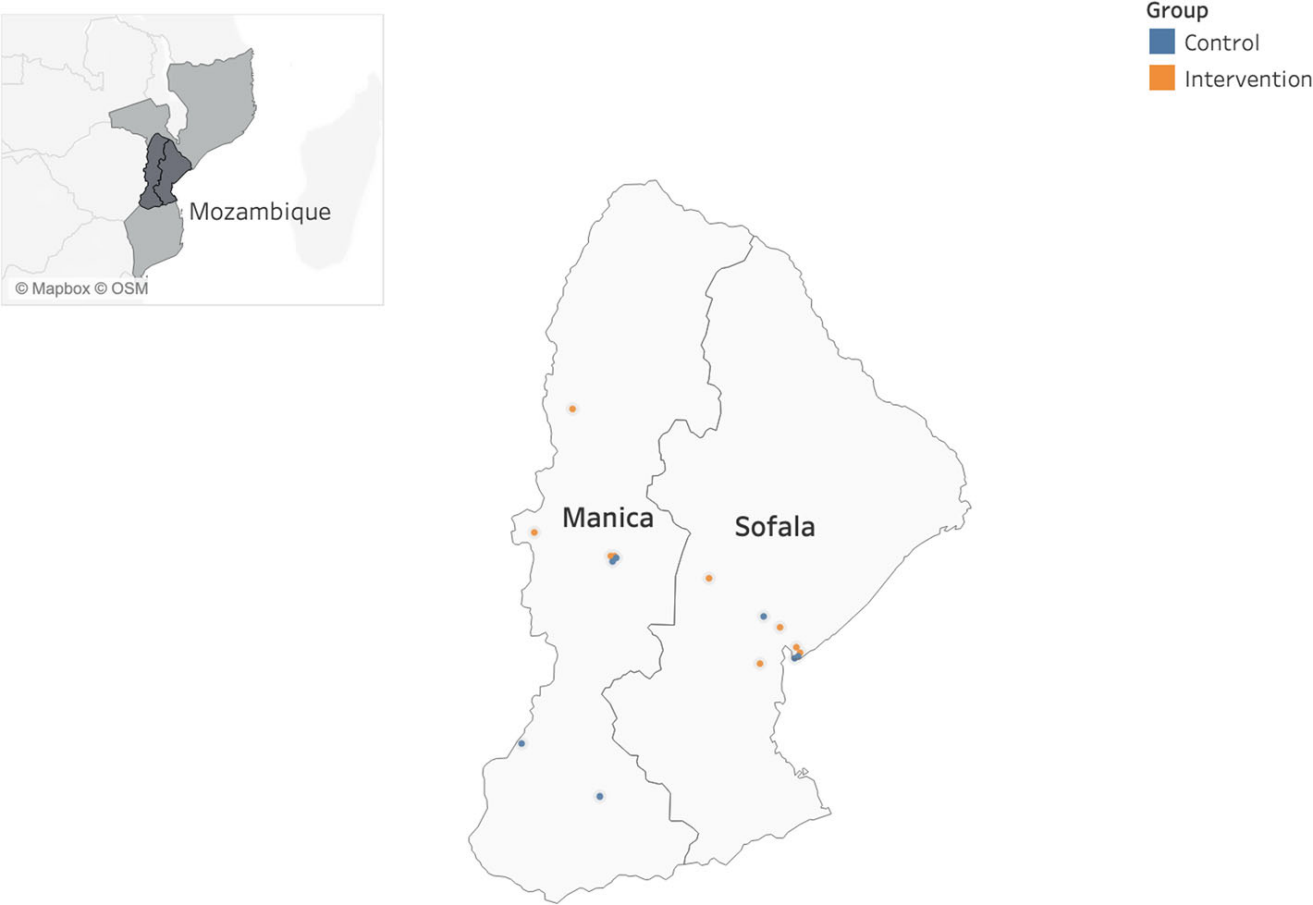
Map of the study area (Manica and Sofala Provinces, Mozambique)

Manica and Sofala provinces (population ~ 4 million; Fig. 3) have higher adult HIV prevalence than the national average (15.3% and 15.5% respectively, compared to 11.5% nationally) [44]. These provinces were selected because of the deep relationship between investigators and health authorities, and absence of structural interventions for hypertension diagnosis and management within HIV care. In central Mozambique, over 98% of formal health services are offered through the public sector [45], and primary care utilization is high, supporting spread and likelihood of population-level impact for a supply-side intervention delivered through the HIV treatment platform.

HIV treatment services have been scaled up in most sub-Saharan countries, including Mozambique, where over 1300 health facilities (81% of public sector health facilities in the country) offer chronic HIV treatment in adult ambulatory services [46]. In this total population of 28 million, more than 850,000 patients are accessing ART [47, 48]. In 2007, Mozambique integrated adult HIV care into general ambulatory services to improve efficiency, reduce stigma, and leverage HIV investments to strengthen the health system. Integrating hypertension screening and management in the HIV care system presents an opportunity to reach a large number of at-risk individuals enrolled in chronic care, and provide a scalable model for application in health centers nationwide.

#### Eligibility criteria

To maximize the potential impact of SAIA-HTN, and reflecting the higher burden of hypertension in high volume health facilities, we will select facilities in either provincial or district capitals offering HIV treatment services through ambulatory clinics (Fig. 3). Eligible facilities will be public sector clinics, see a minimum of 2000 total ambulatory consults per month, and be located in central Mozambique. Facilities in which ongoing prospective studies or similar systems analysis and enhancement techniques are being implemented will be excluded from the trial.

#### Randomization

Given these criteria, 29 facilities in Manica and Sofala provinces meet eligibility. From these 29, 16 study facilities will be randomly selected (representing 18 of 25 districts in the two provinces) by the study team. Clinics will then be randomly assigned 1:1 to intervention or control using constrained randomization to balance province, urban/ peri-urban, total number of PLHIV, and providers by arm [49]. All adult PLHIV (> 14 years) will contribute outcomes data via medical records and registries in ambulatory care. Clients will not be directly enrolled as participants in this study.

### SAIA-HTN impact assessment

Through a mentored process of solution identification and testing, we hypothesize that SAIA-HTN will lead to rapid and sustainable improvements in hypertension service delivery in the eight intervention clinics, assessed via routinely available process indicators and patient-level outcome measures.

#### Study population

All PLHIV-adults (> 14 years) accessing ambulatory care services at study facilities during the study period, including those diagnosed during the consult as well as those diagnosed prior to presentation at care.

#### Exposure definition

Facilities will be considered unexposed prior to the initiation of the SAIA-HTN intervention in their health facility and exposed thereafter. Individuals’ exposure to the intervention will be based on the exposure status of the facility in the calendar month in which they enter care (including those newly identified as hypertensive and those already diagnosed and on an anti-hypertensive medication).

#### Outcomes

The primary SAIA-HTN study process outcome is hypertension screening among PLHIV in ambulatory care services, while the primary clinical outcome is controlled hypertension. Secondary outcomes will include additional quantitative measures that reflect successful progression through steps in the hypertension cascade for PLHIV, as well as HIV-related clinical outcomes (Table 2). Process measures were identified for inclusion as study outcomes because they are sensitive to system-level improvements; represent steps that, if changed, would meaningfully alter patterns of hypertension-related morbidity; and are efficient to collect and readily understood by facility managers and frontline staff. Process measures will be assessed monthly over the study period (including 3 months of pre-intervention baseline measures, 24 months during the intensive intervention phase, and 12 months during the sustainment phase).

Clinical outcomes were included to determine the effect of SAIA-HTN on meaningful hypertension and HIV control over time, which are more dependent on patient behavior and characteristics and may be less sensitive to systems-level improvements.

#### Data sources

Patient-level hypertension and HIV clinical outcomes will be sourced from existing Ministry of Health patient level forms with slight modifications designed to capture the entire hypertension care cascade for PLHIV from screening to controlled hypertension. Data will be abstracted at least weekly into a study database via a RedCAP questionnaire on tablets by study personnel. As part of routine care, each PLHIV patient is assigned a unique identification number that links across service points and clinics, which will be used to abstract registry data for study outcome measures. The database will generate on-demand reports with monthly indicators to populate the HCAT.

#### Power and sample size

Power calculations are based on the primary process outcome of the proportion of ambulatory care visits by PLHIV at which blood pressure is measured, comparing the intervention and control facilities during the final 3 months of the intensive intervention period. Blood pressure screening was selected as the primary outcome as existing guidelines call for it to be routinized into outpatient services for all patients, and it affects the greatest number of PLHIV. Minimum detectable difference calculations were conducted in Stata 14.2 (StataCorp, College Station, Texas), based on a range of plausible values for endline proportion of visits in the control arm and intracluster correlation coefficient (Table 3). Calculations assumed α = 0.05 and 1000 visits by PLHIV patients over each 3-month period. At the start of the pilot study, less than 10% of routine care visits in the pilot facilities included blood pressure screening; in control facilities, we anticipate there will be minimal improvement in screening coverage during the study period (< 5%). Given these parameters, eight intervention and eight control facilities, we will have 80% power to detect an absolute difference in the endline proportion of visits with blood pressure screening between 15 and 29%, depending on endline proportion in the control facilities and intracluster correlation. Given the 31% increase observed in the pilot study [38], we believe this estimated effect size is reasonable.

**Table 3:** Minimum detectable alternative intracluster

	Intracluster correlation (ρ)			
Endline proportion of control arm visits (p0)		0.10	0.15	0.2
	0.05	0.20	0.25	0.29
	0.10	0.28	0.33	0.37
	0.15	0.35	0.40	0.44

The minimum detectable alternative proportion of visits by people living with HIV at which blood pressure is measured during the final 3 months of the intervention, by endline proportion of visits in the control arm and intracluster correlation. Calculations assume α = 0.05. 1000 visits per facility and 80% power

#### Data analysis

Impact analysis will include generalized linear mixed modeling comparing study outcomes between intervention and control facilities (including the proportion of routine visits at which blood pressure is measured; treatment initiation among those diagnosed with hypertension; medication pickup; and hypertension control among those diagnosed by arm). Models will account for clustering by health facility and will assess the impact of adjustment for patient-level covariates (e.g., age, sex, new vs. existing HIV diagnosis) as well as facility-level covariates (e.g., patient volume, staffing levels), and time (in months since study initiation). Secondary analyses will test for interaction between arm and time, and between arm and study phase (pre-intervention, 24-month intervention, and 12-month sustainment periods). We will also conduct a controlled, segmented time-series analysis that incorporates monthly facility-level estimates from the entire 36-month study period (segmented into 3-month pre-intervention, 24-month intervention, and 12-month sustainment periods). This secondary analysis will allow us to fully use available data to assess intervention impact, address both serial and intra-class correlation, and assess temporal patterns in study outcomes.

### Determining drivers of SAIA-HTN intervention implementation heterogeneity

We will apply four implementation science theories, models, and frameworks to examine the implementation process, focusing on clinics as the organizational level.

#### Organizational readiness for implementing change

Organizational readiness for implementing change (ORIC) assesses the extent to which organizational members are psychologically and behaviorally prepared to implement organizational change, which affects decisions to adopt implementation strategies like SAIA-HTN [50, 51]. Readiness includes (1) change commitment, reflecting shared resolve to implement a change, and (2) change efficacy, reflecting a shared belief in the collective capability to implement change. To understand readiness for adopting SAIA-HTN, we will apply the ORIC assessment scale which has been translated, adapted, and implemented to this context as part of the SAIA-SCALE study prior to launching SAIA-HTN [40]. We will apply the ORIC to eight management team members of each intervention district (*n* = 48) and eight health workers per intervention facility (*n* = 64). Analysis will test whether sufficient inter-rater reliability and inter-rater agreement exist to aggregate individual responses to the facility level. If tests do not justify aggregation, we will use a measure of intra-facility variability in readiness rather than a facility-level mean in our analysis. The resulting analysis will provide readiness profiles for each facility as they initiate implementation, which will complement adoption, implementation, and effectiveness data in understanding the broad impact of SAIA-HTN.

#### Facility-level structural readiness assessments

Standardized readiness assessments, adapted from the SARA tool [52] and used in previous SAIA studies, will be carried out annually in all 16 study facilities to assess structural readiness to deliver hypertension services (staffing levels, attributes and training; availability of essential commodities, equipment, and supplies; and infrastructure). Recurrent measurement of structural readiness will inform guidance on essential structural needs to implement SAIA-HTN.

#### Measuring implementation dose

We have developed an approach to measure implementation dose adapted from McHugh et al.’s dimensions of implementation for SAIA-HTN [53]. In this definition of dose, the quantity and quality of both the intervention and participation is considered, recognizing that characteristics beyond quantity may influence effectiveness [54]. This information is captured through both reporting tools and focus group discussions (FGDs) and in-depth interviews (IDIs) (Table 4). Reporting tools will be used to capture the quantity, exposure, reach, duration, and elements of quality of implementation across facilities, using internal program reports, field staff observations, supervision reports, archived facility-level process maps, and quality improvement plans adapted from the original SAIA and refined during the SAIA-HTN pilot. FGD/IDIs will be used to collect qualitative dimensions of implementation dose including intensity, scope, engagement, and features of quality. We will quantify associations between implementation dose and SAIA-HTN cascade improvements to identify best practices, including facility structural characteristics (e.g., staffing levels and experience, patient volume, facility infrastructure, availability of essential drugs, supplies, equipment, etc.). Bivariate analysis and multivariate models will detect associations, specifically correlation between (1) organizational readiness and dose and (2) process/clinical outcomes and dose [55, 56].

**Table 4:** Dimensions and reporting of implementation dose

Intervention dose dimensions	Description	Measurement	
		Intervention period	Sustainment period
Quantity	# CQI micro-interventions by facility	Reports	Reports
Exposure	# TA visits to facilities	Reports	Reports
Intensity	Depth of use of the SAIA-HTN at the facility	FGD/IDI	FGD/IDI
Scope	Breadth of use of SAIA-HTN at the facility (comprehensiveness)	FGD/IDI	FGD/IDI
Reach	# Health workers/patients touched by the intervention	Reports	Reports
Engagement	Commitment/seriousness of staff participating in the intervention	FGD/IDI	FGD/IDI
Duration	Amount of time SAIA-HTN was actively used within a site	Reports	Reports
Quality	Quality of SAIA-HTN implementation over time (complete reporting, iterative cycles, data use)	Reports and FGD/IDI	Reports and FGD/IDI

*CQI* continuous quality improvement, *TA* technical advisor; *FGD* focus group discussions, *IDI* in-depth interviews

#### Consolidated Framework for Implementation Research

We will use the Consolidated Framework for Implementation Research (CFIR) to guide an in-depth examination of the implementation process, define SAIA-HTN core elements, and describe determinants of success and failure across implementing facilities. During the SAIA-HTN pilot, we adapted CFIR tools (http://cfirguide.org/; see Table 5 for constructs of interest) to develop semi-structured topic guides to collect data about select constructs from each of the five CFIR domains, supplemented by targeted elicitation of satisfaction with tools/protocols, intent-to-continue-use, and deviations from the 5-step SAIA protocol. After the completion of the intensive intervention phase (year 3), we will conduct FGDs in each intervention facility with 7–10 clinic staff (sufficient to generate conversation without being too large to become intimidating) (*n* = 56–80) participating in the SAIA-HTN [57]. Facilities will be classified as either high or low performing (identified by facilities’ fidelity to the SAIA-HTN intervention, defined as the number and frequency of SAIA-HTN cycles conducted, and the consistent use of quantitative data to inform progress) [58]. By purposively holding separate FGDs for facilities, and classifying them by high and low implementation fidelity, we intend to uncover salient features of successful implementation.

**Table 5:** CFIR constructs of interest

**I. Intervention characteristics**	
Intervention Source	1
Evidence Strength & Quality	1
Relative Advanatage	1
Adaptability	1
Trialability	1
Complexity	1
Design Quality & Packaging	1
Cost	2
**II. Outer setting**	
Patient Needs & Resources	^
Cosmopolitanism	^
Peer Pressure	1
External Policy & Incentives	1
**III. Inner setting**	
Structural Characteristics	2
Networks & Communication	1
Culture	1
Implementation Climate	1
Readiness for Implementation	1
**IV. Characteristics of individuals**	
Knowledge & Beliefs about the Intervention	1
Self-efficacy	1
Individual Stage of Change	1
Individual Identification with Organization	1
Other Personal Attributes	1
**V. Process**	
Planning	^
Engaging	1
Executing	2
Reflecting & Evaluating	1

1 = qualitative data, 2 = quantitative data, ^ = no primary data collection planned

Additional IDIs will be held with facility (*n* = 24) and district (*n* = 12) managers who participate in the SAIA-HTN trial to collect potentially sensitive information that staff members might be hesitant to share in group settings, such as opinions that lower ranking staff feel uncomfortable sharing with their superiors, or sensitive issues related to staffing that higher ranking staff members feel uncomfortable discussing with lower-ranking team members (e.g., leadership engagement or organizational culture). The IDIs will allow for exploration of the individual experience with disseminating and implementing SAIA-HTN, and capture intervention adaptations over time, such as staff attitudes or identification with the organization. All FGD and IDI participants will be purposively selected by the study staff, to ensure balance of representation across service location and roles. To understand the implementation process and adaptation during the sustainment phase, we will repeat an equal number of FGDs and IDIs at the end of the sustainment period (year 4), using the same sampling scheme as described above. In addition, implementation tracking (reporting on training and supervision) will be conducted to quantify the executing construct within the implementation process domain.

FGDs and IDIs will be conducted in Portuguese by an experienced facilitator accompanied by a note-taker (FGDs only), audio-recorded, transcribed verbatim into Portuguese by trained Mozambican staff, and translated into English by staff fluent in both language. First, two coders in a stepwise, iterative process will code the IDI and FGD transcripts, and will conduct content analysis within a deductive framework to identify key implementation themes (using selected CFIR constructs, but allowing flexibility for other themes to emerge). Coding will be compared across pairs and differences discussed prior to final coding. Case memos will be written and three analysts will assign ratings for each construct. Using a rating process previously applied to the CFIR [36, 59], ratings will reflect the positive or negative influence (valence) and the strength of each construct. Constructs will be coded as missing too much data (M), not (0), weakly (+ 1/− 1), or strongly (+ 2/− 2) distinguishing low/high performance. Findings will be used to develop recommendations for SAIA-HTN implementation, including its core components, intervention adaptions, and lessons learned.

### SAIA-HTN economic evaluation

We will estimate total incremental and unit costs of integrating hypertension diagnosis and management into HIV care. Pending effectiveness, a cost-utility analysis to measure the cost per additional disability adjusted life years (DALY) will be conducted.

#### Cost estimation

A payer perspective (e.g., Mozambican Ministry of Health) will be used for the analyses (Table 6). Since implementation of SAIA-HTN is integrated into the healthcare system, we will estimate the incremental costs of introducing SAIA-HTN to the existing system. Activity-based cost menus will identify all start-up and recurrent activities and measure resource use and costs from intervention design through implementation. Personnel time for SAIA-HTN tasks will be estimated based on activity tracking and surveys administered to study nurses and district supervisors. We will supplement these data with information from the SAIA-HTN expense reports for human resources, training, supplies, intervention delivery, and facility support for workflow modifications, etc. Cost metrics will include the total incremental costs of the SAIA-HTN intervention and average unit costs, including cost per PLHIV adult (1) screened for HTN, (2) diagnosed with HTN, (3) initiated on HTN medications, (4) adherent to anti-HTN medications (measured via timely medication pick up), (5) maintained controlled HTN, and (6) with HIV viral load suppressed. We will model potential cost-offsets due to the impact of SAIA-HTN on reduced outpatient visits and hospitalizations within the study, using Mozambique unit cost estimates from the WHOCHOICE database [60].

**Table 6:** SAIA-HTN economic evaluation summary

Perspective	Payer (MOH) to determine incremental costs/net benefits of integrating SAIA-HTN into HIV chronic care. Societal to determine the incremental cost and benefits of integrating SAIA-HTN into HIV chronic care.
Cost estimates	Intervention costs (SAIA-HTN delivery), medical costs averted and accrued of additional hypertensive PLHIV diagnosed with HTN, initiated on anti-hypertensive treatment, and retained in care.
Cost data collection	Facility-level cost data collection on activities and resource use, including time motion studies for personnel time. Additional information collected on expenses from the SAIA-HTN budget, published secondary data on government information on civil servant salary costs, and medical supplies.
Primary outcomes	Proportion of PLHIV patients 1) screened for HTN, 2) diagnosed with HTN, 3) initiating hypertension medications, 4) adherent to hypertension medications (via timely medication pick up), and 5) with controlled HTN, and DALYs averted.
Discounting	A discounting rate of 3% will be used, and varied from 0% to 5% in sensitivity analyses [66].
Analytic time frame	Using mathematical models estimating medium and long-term health outcomes, the ICER for progression through the HTN cascade for PLHIV and DALY averted will be reported over 1, 5, 10-, and 15-year time frames.

#### Modeling outcomes

Mathematical models will simulate the SAIA-HTN intervention to dynamically evaluate the population-level impact of the intervention. We will parameterize the model using a combination of study data (e.g., clinical utilization and outcomes) and estimates from the literature. Model outcomes will include deaths averted due to cardiovascular and cerebrovascular events, and DALYs averted. We will conduct sensitivity analyses to account for uncertainty in key parameters. We will also model the impact of adhering to regional (PASCAR) guidelines for hypertension management for a national cohort of adults in Mozambique and validate the model using Mozambique’s stepwise approach to surveillance (STEPS) survey data on hypertension prevalence [7], as well as any other models as they are identified.

#### Economic evaluation

Cost and outcomes comparing SAIA-HTN with the *status quo* (obtained from the aforementioned analyses) will be combined to calculate the incremental cost effectiveness ratio (ICER) defined as the incremental cost per death or disability adjusted life year (DALY) averted. We will use the study effectiveness data to estimate the net deaths averted due to cardiovascular and cerebrovascular events. We will estimate the number of DALYs averted to capture the gap between current and ideal health, using study outcomes and assumptions reflecting the Mozambique context (i.e., life expectancy, average duration of illness, etc.) [61]. Following WHO guidelines [62], and to facilitate comparisons with other strategies to guide resource allocation, SAIA-HTN will be considered cost-effective, after situating both the costs and effectiveness within the Mozambican context, which will include consideration of the disease burden and the budget of this setting [63, 64]. Analysis will be from the *payer perspective*, taking into consideration productivity and health system costs, as well as future health system costs averted due to prevention of hypertension in PLHIV.

### Trial status

Preparations for SAIA-HTN initiated in May 2019. Initiation of the SAIA-HTN trial is planned for June 2020.

## Discussion

SAIA-HTN is a pragmatic trial to test a novel, scalable approach to optimize hypertension diagnosis and management in PLHIV. The implementation approach aligns with existing administrative structures, providing a practical tool to integrate into routine functions of district and facility supervisors. SAIA uses systems engineering tools to visualize and quantify interconnected service delivery steps, allowing health workers to prioritize interventions that can move more individuals through critical care steps in their clinic. SAIA is flexible and adaptable to local settings, and empowers health care workers to test their ideas for optimization through a series of “micro-changes.” Rather than testing a single strategy that may become irrelevant after policy or technology changes, SAIA has longevity, as the approach is adaptable to the changing service landscape, which increasingly includes co-morbid conditions like hypertension.

Our design includes multiple novel implementation science methods. The CFIR provides a guide for intervention planning, implementation, and by addressing “what works, where and why”; identifies local barriers to implementation; and contributes to the knowledge base around implementation of interventions across diverse settings [59]. Assessing readiness for change identifies organizational-level determinants of adoption and implementation of interventions. We will use the organizational readiness for implementing change (ORIC) scale to assess facility readiness for SAIA-HTN adoption [50, 51]. Cost-effective analyses rarely assess CVD management strategies among PLHIV, and more research is needed [65]. This study will model long-term outcomes (DALYs) to capture the minimum data set for economic evaluation recently developed for researchers studying HIV/NCD integration [65]. State of the art implementation science methods strengthen our evaluative framework for multi-level, theory-based adaptation of interventions, and exploits outcome heterogeneity to assess implementation barriers and facilitators.

## Supplementary information

**Additional File 1:** CONSORT Checklist Facility eligibility and randomization

## Abbreviations

ART: Anti-retroviral therapy
CFIR: Consolidated Framework for Implementation Research
CQI: Continuous quality improvement
CVD: Cardiovascular disease
DALY: Disability adjusted life years
FGD: Focus group discussion
HCAT: Hypertension cascade analysis tool
HIV: Human immunodeficiency virus
HTN: Hypertension
ICER: Incremental cost effectiveness ratio
IDI: In-depth interview
LMIC: Low- and middle-income countries
NCD: Non-communicable disease
ORIC: Organizational readiness for implementing change
PASCAR: Pan-African Society of Cardiology
PLHIV: People living with HIV
SAIA: Systems analysis and improvement approach
SOP: Standard operating procedures
sSA: Sub-Saharan Africa
STEPS: Stepwise approach to surveillance
