# Correction to: Systems analysis and improvement approach to optimize the hypertension diagnosis and care cascade for PLHIV individuals (SAIA-HTN): a hybrid type III cluster randomized trial

**DOI:** 10.1186/s13012-020-00980-6

**Published:** 2020-03-19

**Authors:** Sarah Gimbel, Ana Olga Mocumbi, Kristjana Ásbjörnsdóttir, Joana Coutinho, Leonel Andela, Bonifacio Cebola, Heidi Craine, Jonny Crocker, Leecreesha Hicks, Sarah Holte, Rodrigues Hossieke, Edgar Itai, Carol Levin, Nelia Manaca, Filipe Murgorgo, Miguel Nhumba, James Pfeiffer, Isaias Ramiro, Keshet Ronen, Nona Sotoodehnia, Onei Uetela, Anjuli Wagner, Bryan J. Weiner, Kenneth Sherr

**Affiliations:** 1 Department of Child, Family and Population Health Nursing, University of Washington School of Nursing, 1959 NE Pacific St, Seattle, WA 98195 USA; 2 Department of Global Health, University of Washington Schools of Medicine and Public Health, 1705 NE Pacific St, Seattle, WA 98195 USA; 3 Health Alliance International (HAI), 1107 NE 45th St, Suite 350, Seattle, WA 98105 USA; 4 Faculty of Medicine, Eduardo Mondlane University, Avenida Salvador Allende, 702 Maputo, Mozambique; 5 Health Alliance International, Caixa Postal, #23 Maputo, Mozambique; 6 Department of Epidemiology, University of Washington School of Public Health, 1705 NE Pacific St, Seattle, WA 98195 USA; 7 Beira Central Hospital, Beira, Mozambique; 8 Manica Provincial Health Department, Chimoio, Mozambique; 9 Sofala Provincial Health Department, Beira, Mozambique; 10 Department of Cardiology, University of Washington School of Medicine, 325 9th Avenue, Seattle, WA 98104 USA

**Correction to: Implementation Sci**

**https://doi.org/10.1186/s13012-020-0973-4**

Following publication of the original article [1], the authors reported a typo in the title of the article. In this Correction the incorrect and correct title are shown, with the correction shown in bold. The original article has been corrected.

Originally the title was published as:

Systems analysis and improvement approach to optimize the hypertension diagnosis and case cascade for PLHIV individuals (SAIA-HTN): a hybrid type III cluster randomized trial

The correct title is:

Systems analysis and improvement approach to optimize the hypertension diagnosis and **care** cascade for PLHIV individuals (SAIA-HTN): a hybrid type III cluster randomized trial
