# Supplementary material for: Systems analysis and improvement approach to optimize the hypertension diagnosis and case cascade for PLHIV individuals (SAIA-HTN): a hybrid type III cluster randomized trial
Source: Implement Sci. 2020 Mar 6;15:15. doi: 10.1186/s13012-020-0973-4 (PMC7059349; doi:10.1186/s13012-020-0973-4)
Supplement: Supplementary file 1 — Additional File 1: CONSORT Checklist Facility eligibility and randomization [file 13012_2020_973_MOESM1_ESM.pdf]

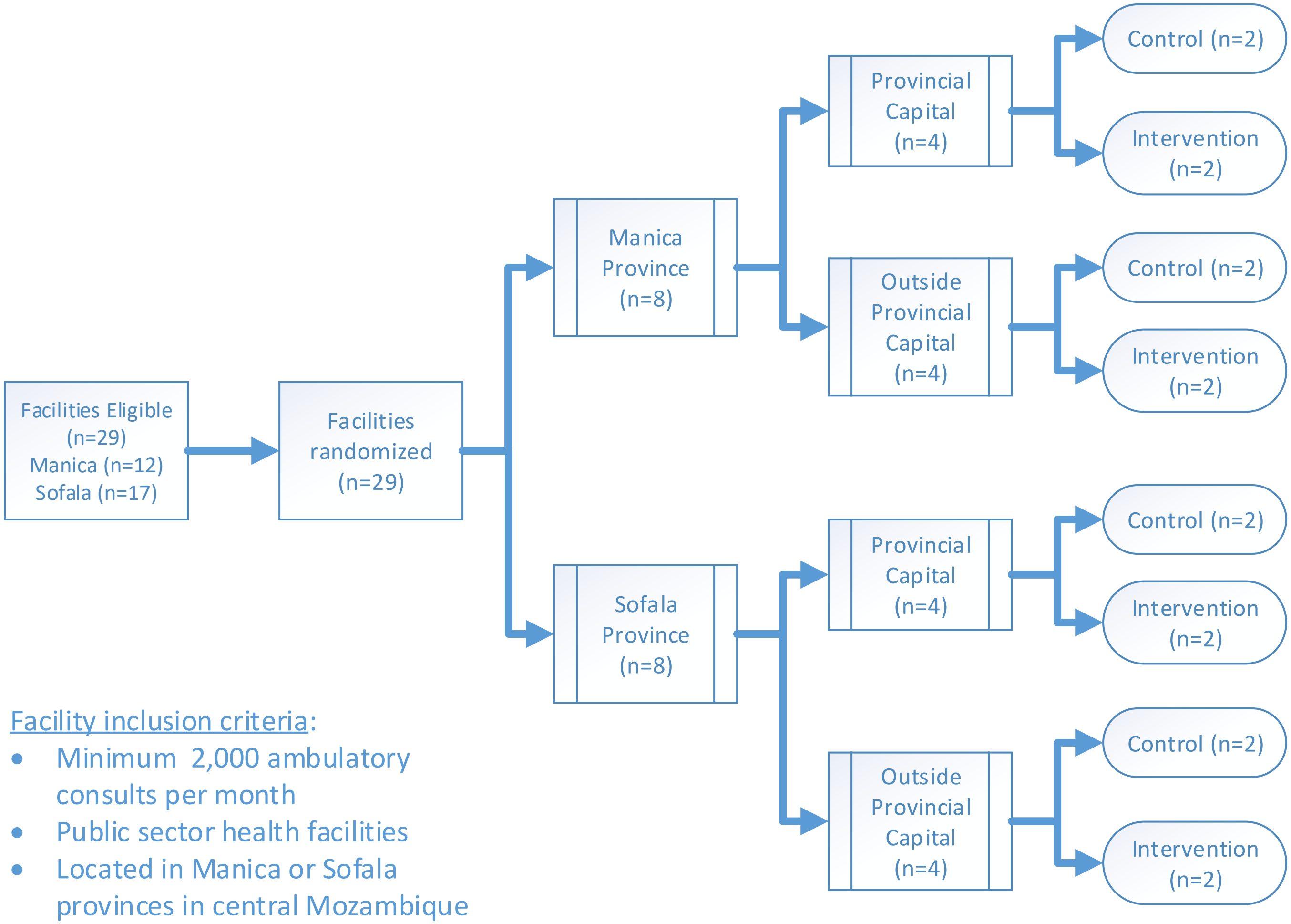

Facility inclusion criteria:

- Minimum 2,000 ambulatory consults per month
- Public sector health facilities
- Located in Manica or Sofala provinces in central Mozambique
- No ongoing ongoing prospective studies or similar systems analysis and enhancement techniques being implemented in ambulatory care

Additional File 1: CONSORT Checklist

Facility eligibility and randomization
